# Predicting RNA-binding residues from evolutionary information and sequence conservation

**DOI:** 10.1186/1471-2164-11-S4-S2

**Published:** 2010-12-02

**Authors:** Yu-Feng Huang, Li-Yuan Chiu, Chun-Chin Huang, Chien-Kang Huang

**Affiliations:** 1 Department of Computer Science and Information Engineering, National Taiwan University, Taipei, Taiwan, Republic of China; 2 Department of Engineering Science and Oceanic Engineering, National Taiwan University, Taipei, Taiwan, Republic of China

## Abstract

**Abstract:**

**Background**

RNA-binding proteins (RBPs) play crucial roles in post-transcriptional control of RNA. RBPs are designed to efficiently recognize specific RNA sequences after it is derived from the DNA sequence. To satisfy diverse functional requirements, RNA binding proteins are composed of multiple blocks of RNA-binding domains (RBDs) presented in various structural arrangements to provide versatile functions. The ability to computationally predict RNA-binding residues in a RNA-binding protein can help biologists reveal important site-directed mutagenesis in wet-lab experiments.

**Results**

The proposed prediction framework named “ProteRNA” combines a SVM-based classifier with conserved residue discovery by WildSpan to identify the residues that interact with RNA in a RNA-binding protein. Although these conserved residues can be either functionally conserved residues or structurally conserved residues, they provide clues on the important residues in a protein sequence. In the independent testing dataset, ProteRNA has been able to deliver overall accuracy of 89.78%, MCC of 0.2628, F-score of 0.3075, and F_0.5_-score of 0.3546.

**Conclusions**

This article presents the design of a sequence-based predictor aiming to identify the RNA-binding residues in a RNA-binding protein by combining machine learning and pattern mining approaches. RNA-binding proteins have diverse functions while interacting with different categories of RNAs because these proteins are composed of multiple copies of RNA-binding domains presented in various structural arrangements to expand the functional repertoire of RNA-binding proteins. Furthermore, predicting RNA-binding residues in a RNA-binding protein can help biologists reveal important site-directed mutagenesis in wet-lab experiments.

## Background

RNA-binding proteins (RBPs) are designed to efficiently recognize specific RNA sequences after they are derived from the DNA sequences. Protein-RNA interactions are fundamental to cellular processes, including the assembly and function of ribonucleoprotein particles (RNPs), such as ribosomes and spliceosomes and the post-transcriptional regulation of gene products. For satisfying diverse functional requirements, RNA binding proteins are composed of multiple blocks of RNA-binding domains (RBDs) presented in various structural arrangements to provide versatile functionality [1,2]. Although RNA structure is hierarchical, that is, the primary sequence determines the secondary structure which, in turns, determines tertiary structure, the tertiary structure of RNA is not as stable as secondary structure and is hard to predict [3]. However, sequence conservations in RNA-binding domains have been discovered in RNA-binding proteins [4, 5, 6, 7]. With the recent growth of protein-RNA complexes in the Protein Data Bank (PDB) [8] and the Nucleic Acid Database (NDB) [9], structural analysis on RNA-binding pockets [10, 11, 12, 13, 14, 15, 16, 17, 18] and the themes of RNA-protein recognition [18, 19, 20] have been investigated as well.

Most recent works on predicting RNA-binding residues used support vector machine (SVM) with protein evolutionary information from protein sequence. Wang and Brown (2006) developed the web service, BindN [21], to predict DNA and RNA binding sites using sequence features to represent structural characteristics including relative solvent accessible surface area, side chain pKa, hydrophobicity index and molecular mass of an amino acid. Tong *et al.* (2008) [22] proposed the hybrid RISP (RNA-Interaction Site Prediction) method by adjusting cutoff value of SVM discrimination function to improve prediction performance. Kumar *et al.* (2008) developed Pprint [23] by using evolutionary profiles of the position-specific scoring matrices (PSSMs) and amino acid composition while they also adjusted cutoff value of SVM discrimination function to improve prediction performance. Wang *et al.* (2008) developed PRINTR [24] by using additional structural information from protein-RNA complexes. Cheng et al. (2008) developed RNAProB [25] by smoothing PSSM profiles with consideration of the correlation and dependency from the neighboring residues for each amino acid in a protein. Spriggs et al. (2009) [26] developed the PiRaNhA by using support vector machine with a PSSM profile and three amino acid properties, including interface propensity (IP), predicted solvent accessibility (pA) and hydrophobicity (H) for recognizing RNA-binding residues [27]. Other machine learning approaches such as neural network and Naïve Bayes classifier have also been applied to predict RNA-binding residues. Jeong et al. (2004) [28] applied artificial neural network (ANN)-based method with amino acid sequence and predicted secondary structure information and improved the performance by using post-processing procedures such as state-shifting and filtering isolated interacting residues from prediction. Improved version by Jeong et al. (2006) [29] used evolutionary information extracted from PSI-BLAST profiles and CLUSTALW alignment. Terribilini *et al.* (2006) [30] applied a Naïve Bayes classifier with amino acid sequence information for predicting RNA interacting residues and presented the results through the web service RNABindR [31]. The ability to computationally predict RNA-binding residues in a RNA-binding protein can help biologists reveal site-directed mutagenesis in wet-lab experiments.

Caragea et al. [32] explored the problem of assessing the performance of classifiers trained on macromolecular sequence data, with the emphasis on cross-validation and data selection methods. In comparison of window-based *k*-fold cross-validation and sequence-based *k*-fold cross-validation, window-based cross-validation can yield overly optimistic estimates of the performance of classifier relative to the estimates obtained using sequence-based cross-validation. RNAProB, BindN, RISP, PRINTR and PiRaNhA are predictors that report performance window-based *k*-fold cross-validation while Pprint and RNABindR report performance with sequence-based *k*-fold cross-validation. The predictors evaluated with window-based *k*-fold cross-validation have superior performance than those with sequence-based *k*-fold cross-validation. The reason is that data instances in the testing fold would be predicted by data instances with sub-sequence identity higher than 25% in the training fold in window-based *k*-fold cross-validation. Therefore, in data with class imbalance, the metrics that measure the classification performance must be chosen carefully. Matthew's correlation coefficient (MCC), F-score and F_0.5_-score are widely applied to assess the prediction performance. MCC is used to measure prediction quality with the consideration of both under- and over-predictions. F-score and F_0.5_-score are used to assess balanced prediction quality on both positive class and negative class.

In this article, we proposed the prediction framework “ProteRNA” with the combination of SVM-based classifier with evolutionary profiles and conserved residues discovery by sequence conservation for identifying RNA-interacting residues in a RNA-binding protein. In the SVM-based classifier, we use features including position-specific scoring matrix computed by PSI-BLAST and secondary structure information predicted by PSIPRED as feature vectors [33]. To exploit the sequence conservation information, WildSpan [34] (http://biominer.bime.ntu.edu.tw/wildspan/), which is developed to discover functional signatures and diagnostic patterns of proteins directly from a set of unaligned protein sequences, is incorporated. The most distinguishing feature of WildSpan is that it links short motifs (local conserved regions) with large flexible gaps to deliver the most frequently observed discontinuous patterns present in related proteins. WildSpan has been embedded in many applications [35, 36, 37, 38, 39] to discover functionally important residues; therefore, we apply WildSpan to discover conserved residues as RNA-binding residues in a protein sequence to improve prediction performance on detecting more RNA-binding residues. The independent testing dataset collected for performance evaluation contains 33 testing RNA-binding proteins with less than 30% sequence identity against with training data. In the independent testing dataset, ProteRNA has been able to deliver overall accuracy of 89.78%, MCC of 0.2628, F-score of 0.3075, and F_0.5_-score of 0.3546. We emphasize MCC, F-score and F_0.5_-score because it provides the biochemist with a confidence level for designing an experiment to confirm whether a predicted binding residue is really involved in interaction with the RNA.

## Results and discussion

In this section, we will report the experiments conducted to evaluate the performance of our proposed approach, ProteRNA with the combination of SVM-based classifier with evolutionary profiles and conserved residues discovery by sequence conservation. In order to avoid bias, we repeated 5-fold cross-validation procedure 20 times to observe prediction performance on the training dataset RB147 (see Materials and Methods for details). For each run, we applied sequence-based 5-fold cross-validation; therefore, protein chains will be randomly divided into 5 folds: one fold for testing and remaining 4 folds for training. For this study, LIBSVM (http://www.csie.ntu.edu.tw/~cjlin/libsvm) was used for data training and classification and WildSpan was used for detecting conserved residues from homologous protein sequences. We use independent testing dataset containing 33 protein chains for comparing ProteRNA with other predictors such as PiRaNhA, Pprint, BindN, and PRIP.

### Performance evaluation by five-fold cross-validation

In order to avoid bias, we repeated 5-fold cross-validation 20 times to observe prediction performance and experimental result was shown in Table 1. Only using SVM-based classifier, ProteRNA_SVM_ delivers overall sensitivity of 38.85%, specificity of 97.01%, precision of 75.99%, accuracy of 85.93%, MCC of 0.4732, F-score of 0.5170 and F_0.5_-score of 0.6343. Since the experiments are repeated 20 times for reducing prediction bias, standard deviation for each assessment is also listed. The results have been obtained using the training parameters, C = 2^1^, γ = 2^-5^, which give better results than other values for prediction of RNA-binding residues. Then WildSpan only outputs patterns for each input protein chain once so there is no information about standard deviation for each assessment. ProteRNA_WildSpan_ delivers overall sensitivity of 12.28%, specificity of 96.26%, precision of 43.60%, accuracy of 80.27%, MCC of 0.1489, F-score of 0.1916 and F_0.5_-score of 0.2887. After combining prediction results by ProteRNA_SVM_ and ProteRNA_WildSpan_, ProteRNA delivers overall sensitivity of 44.84%, specificity of 93.56%, precision of 62.10%, accuracy of 84.28%, MCC of 0.4378, F-score of 0.5208 and F_0.5_-score of 0.5766.

**Table 1:** Prediction performance evaluated by the 5-fold cross-validation using the training dataset, RB147

Predictors	Sensitivity	Specificity	Precision	Accuracy	MCC	F-score	F_0.5_-score
ProteRNA_SVM_	38.85% ± 0.46%	97.01% ± 0.09%	75.99% ± 0.48%	85.93% ± 0.08%	0.4732 ± 0.0036	0.5170 ± 0.0040	0.6343 ± 0.0034

ProteRNA_WildSpan_	12.28%	96.26%	43.60%	80.27%	0.1489	0.1916	0.2887

ProteRNA	44.84% ± 0.37%	93.56% ± 0.09%	62.10% ± 0.25%	84.28% ± 0.06%	0.4378 ± 0.0027	0.5208 ± 0.0027	0.5766 ± 0.0022

As reported by Towfic et al. [40], over half (55.7%) of the RNAs are rRNAs in the dataset of RB147. According to their study, Table 2 shows the distribution of different categories of RNAs on RNA-binding residues. rRNA is the major group that contains about 38% positive samples in rRNA group. Remaining groups presents highly imbalanced class dataset, containing about 10% positive sample in average. If the predictor tries to predict all samples as negative class exclusive of rRNA group, the predictor may gain better performance in assessment but provide no clues for biologists. Table 1 describes the average prediction performance of 20 runs of 5-fold cross-validation; however, we only choose one of the repeated experiments that had a performance that is close to the average performance for detailed analysis in Table 3. As shown in Table 3 ProteRNA_WildSpan_ predicts an equal amount of true positives and false positives on average. Previous research on studying RNA-binding domains revealed that RNA binding proteins are composed of multiple blocks of RNA-binding domains to provide versatile functionality. Therefore, conserved residues in the same RNA-binding domain from different RNA-binding proteins would not always interact with a specific RNA. Furthermore, while combining prediction results predicted by ProteRNA_SVM_ and ProteRNA_WildSpan_, ProteRNA_WildSpan_ detected additional RNA-binding residues that ProteRNA_SVM_ didn’t predict.

**Table 2:** Statistical information of the training dataset, RB147 in terms of RNA-binding residues

	Number of RNA-binding residues	Total number of residues	Ratio of RNA-binding residues
rRNA	3916	10267	38.14%
mRNA	256	1878	13.63%
tRNA	1230	12401	9.92%
others	755	7778	9.71%

Total	6157	32324	19.05%

**Table 3:** Prediction performance breakdown in terms of the categories of RNA using the training dataset, RB147

Predictor	RNA	TP	FP	TN	FN	Sensitivity	Specificity	Precision	Accuracy	MCC	F-score	F_0.5_-score
ProteRNA_SVM_	rRNA	2060	537	5814	1856	52.60%	91.54%	79.32%	76.69%	0.4933	0.6326	0.7201
	mRNA	27	16	1606	229	10.55%	99.01%	62.79%	86.95%	0.2193	0.1806	0.3154
	tRNA	234	171	11000	996	19.02%	98.47%	57.78%	90.59%	0.2942	0.2862	0.4105
	others	109	93	6930	646	14.44%	98.68%	53.96%	90.50%	0.2441	0.2278	0.3487

	Total	2430	823	25344	3727	39.47%	96.86%	74.70%	85.92%	0.4741	0.5165	0.6338
ProteRNA_WildSpan_	rRNA	554	412	5939	3362	14.15%	93.51%	57.35%	63.24%	0.1274	0.2270	0.3560
	mRNA	67	121	1501	189	26.17%	92.54%	35.64%	83.49%	0.2139	0.3018	0.3323
	tRNA	50	173	10998	1180	4.07%	98.45%	22.42%	89.09%	0.0566	0.0688	0.1178
	others	85	272	6751	670	11.26%	96.13%	23.81%	87.89%	0.1045	0.1529	0.1947

	Total	756	978	25189	5401	12.28%	96.26%	43.60%	80.27%	0.1489	0.1916	0.2887
ProteRNA	rRNA	2256	878	5473	1660	57.61%	86.18%	71.98%	75.28%	0.4618	0.6400	0.6856
	mRNA	89	138	1484	167	34.77%	91.49%	39.21%	83.76%	0.2764	0.3685	0.3823
	tRNA	238	304	10867	992	19.35%	97.28%	43.91%	89.55%	0.2431	0.2686	0.3502
	others	177	366	6657	578	23.44%	94.79%	32.60%	87.86%	0.2118	0.2727	0.3024
	Total	2760	1686	24481	3397	44.83%	93.56%	62.08%	84.28%	0.4376	0.5206	0.5764

As we known, rRNA is the major group among the training dataset. Comparing the amount of RNA-binding proteins in terms of interacting target (*e.g.* rRNA, tRNA, mRNA), we find that tRNA generally has the most interaction partners followed by mRNA and rRNA has the least partners. ProteRNA_SVM_ tends to predict negative for proteins in the mRNA group and over-predict either positive class or negative class in tRNA group. However, ProteRNA_WildSpan_ shows no different between categories of RNAs because of discovered homologous proteins in Swiss-Prot. In addition, ProteRNA_WildSpan_ detects conserved residues as binding residues that cover regions that ProteRNA_SVM_ doesn’t predict; therefore, we apply WildSpan to detect conserved residues because these conserved residues have higher probability to play roles in interacting RNAs.

### Comparison with other predictors by independent testing

Only predictors that predict RNA-binding residues from protein primary sequence information were selected for performance comparison. In addition, RISP did not respond with any prediction results after submitting the jobs and PRINTR is unavailable. According to the designed framework of RNABindR, if there is an exact matched protein chain in Protein Data Bank, RNABindR will return the actual RNA-binding residues of protein-RNA complex. Therefore, it is difficult to distinguish whether the returned result is actually binding or predicted binding so RNABindR will be excluded. Finally, Table 4 shows the prediction performance of ProteRNA in comparison with PiRaNhA, Pprint, BindN, and PRIP. While ordering prediction performance in terms of MCC, ProteRNA delivers better performance than other predictors in accuracy, MCC, and F_0.5_-score.

**Table 4:** Comparison of ProteRNA with other predictors using the independent testing dataset, RB33

Predictor*	TP	FP	TN	FN	Sensitivity	Specificity	Precision	Accuracy	MCC	F-score	F_0.5_-score
ProteRNA	222	340	8563	660	25.17%	96.18%	39.50%	89.78%	0.2628	0.3075	0.3546
PiRaNhA	265	538	8365	617	30.05%	93.96%	33.00%	88.20%	0.2504	0.3145	0.3236
Pprint	447	1782	7121	435	50.68%	79.98%	20.05%	77.34%	0.2094	0.2873	0.2281
BindN	348	1613	7290	534	39.46%	81.88%	17.75%	78.06%	0.1527	0.2449	0.1994
PRIP	131	835	8068	751	14.85%	90.62%	13.56%	83.79%	0.0526	0.1418	0.1380

*Order by MCC.

**Figure 1:**
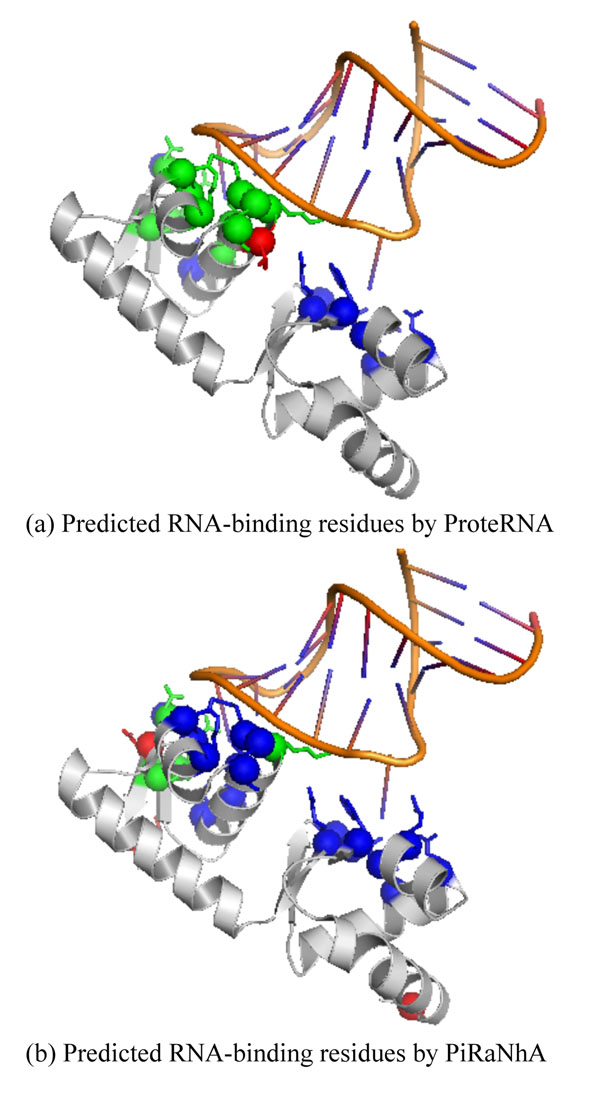
**Case study on *E. coli* SelB (PDBID 2PJPA)** Residues colored by green, red, and blue represent true positive, false positive and false negative, respectively. (a) Predicted RNA-binding residues by ProteRNA. (b) Predicted RNA-binding residues by PiRaNhA.

**Figure 2:**
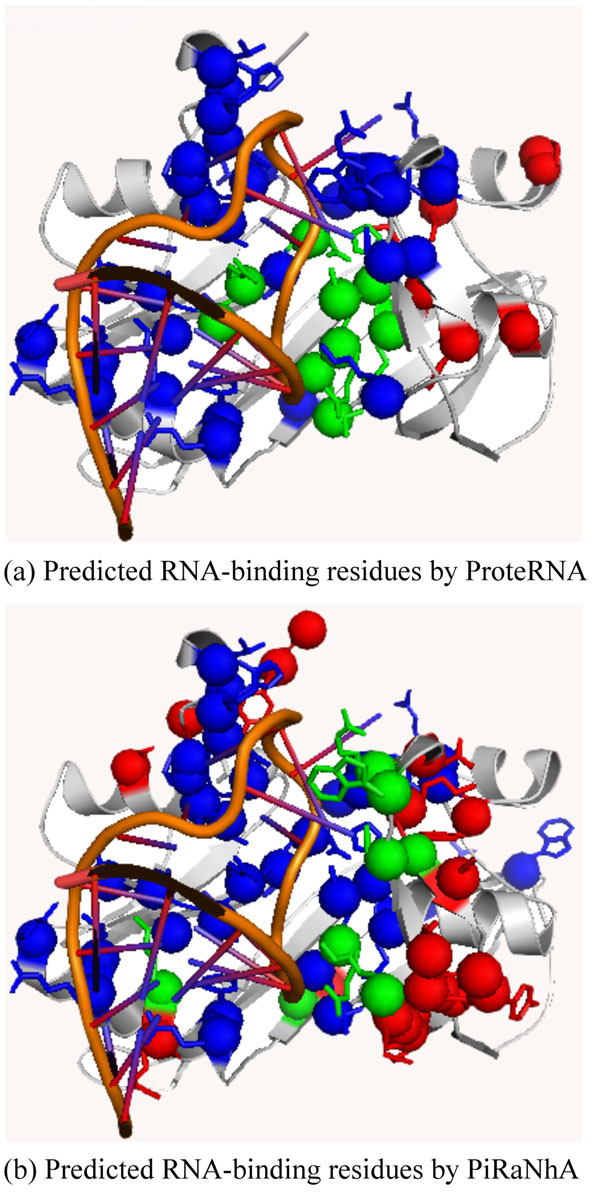
**Case study on RluA (PDBID 2I82C)** Residues colored by green, red, and blue represent true positive, false positive and false negative, respectively. (a) Predicted RNA-binding residues by ProteRNA. (b) Predicted RNA-binding residues by PiRaNhA.

Table 5 shows the Top-10 predictions by different predictors ordered by the MCC and precision among 33 independent testing samples. In terms of MCC, we can find that at least 4 predictors have predictions in 6 protein chains of Top-10 ranking. In terms of precision, we can find that at least 4 predictors have predictions in 7 protein chains of Top-10 ranking. Figure 1 (a) and (b) show the predicted RNA-binding residues in the case of *E. coli* SelB protein with PDB ID 2PJPA [41] by ProteRNA and PiRaNhA respectively. In this case, because WildSpan does not mine any patterns for 2PJPA, only ProteRNA_SVM_ gives prediction result. Figure 2 (a) and (b) show the predicted RNA-binding residues in the case of RluA [42]. In this case, ProteRNA outperforms than other predictors in terms of MCC. BindN and Pprint tend to predict more and more class label for each residue; therefore, they recommend more and more false positives and false negatives. Meanwhile, PRIP and PiRaNhA have similar performance in predicting RNA-binding residues in the case of 2I82C. These figures are rendered by PyMOL (http://www.pymol.org/).

**Table 5:** Comparison of the top 10 ranking predictions with results from other predictors

Rank	ProteRNA	PiRaNhA	Pprint	BindN	PRIP
(a) Rank by MCC

1	2PJP_A	**2QAM_Z**	**2QAM_Z**	**2QAM_Z**	**2PY9_C**
2	**2QAM_Z**	***2QBE_T***	**1VS8_O**	**2PY9_C**	**2QAM_Z**
3	**2PY9_C**	***2DER_B***	2PJP_A	**1VS8_O**	2HYI_D
4	**1VS8_O**	***2G4B_A***	**2PY9_C**	***2QBE_T***	2NQP_B
5	***2G4B_A***	**1VS8_O**	2GYA_3	***2G4B_A***	2IY5_A
6	**2Q66_A**	**2PY9_C**	***2DER_B***	***2DER_B***	**1VS8_O**
7	2I82_C	2G8K_A	***2G4B_A***	2J0Q_A	2I82_C
8	***2DER_B***	2OZB_B	***2QBE_T***	2IPY_B	2V47_C
9	***2QBE_T***	2V47_C	2DR2_A	2HVR_A	2GJE_A
10	2DR2_A	2GJE_D	2QKK_F	2GTT_G	2JEA_B

MCC of Rank 1	0.6668	0.6415	0.6006	0.4364	0.5521

MCC of Rank 10	0.3063	0.2719	0.2390	0.1951	0.0517

(b) Rank by precision

1	2Q66_A	**2GYA_3**	**2GYA_3**	**2QAM_Z**	**2QAM_Z**
2	2PJP_A	***2QBE_T***	**2QAM_Z**	***2QBE_T***	**2PY9_C**
3	**2PY9_C**	**2QAM_Z**	***2QBE_T***	**1VS8_O**	**1VS8_O**
4	**2QAM_Z**	**1VS8_O**	**1VS8_O**	**2PY9_C**	***2QBE_T***
5	***2DER_B***	2OZB_B	**2PY9_C**	**2GYA_3**	***2I82_C***
6	**1VS8_O**	**2PY9_C**	***2DER_B***	***2G4B_A***	***2V47_C***
7	**2GYA_3**	***2DER_B***	***2V47_C***	2J0Q_A	2IY5_A
8	***2I82_C***	***2V47_C***	***2I82_C***	***2I82_C***	**2GYA_3**
9	***2QBE_T***	***2G4B_A***	***2G4B_A***	***2DER_B***	***2G4B_A***
10	2G8K_A	2Q66_A	2GJE_A	***2V47_C***	2GJE_A

Precision of Rank 1	100.00%	100.00%	76.92%	76.47%	75.00%

Precision of Rank 10	50.00%	35.71%	25.00%	24.00%	13.33%

Values in bold indicate listing in the top 10 by at least 5 predictors.

Values in bold and italics indicate listing in the top 10 by at least 4 predictors.

## Conclusions

This article presents the design of a sequence based predictor aiming to identify the RNA-binding residues in a RNA-binding protein by machine learning and pattern mining approaches. RNA-binding proteins play different roles while interacting with different categories of RNAs to represent diverse functions. However, RNA-binding proteins are accommodated by the presence of multiple copies of these RNA-binding domains presented in various structural arrangements to expand the functional repertoire of RNA-binding proteins. Therefore, it is still difficult to predict RNA-binding residues in a RNA-binding protein. Furthermore, predicting RNA-binding residues in a RNA-binding protein can help biologists reveal site-directed mutagenesis in wet-lab experiments.

In the experiments reported in this article, ProteRNA used not only evolutionary profile with predicted secondary structure but also sequence conservation information. Although these conserved residues can be functional conserved residues or structural conserved residues, they also provide clues to indicate the important residues in a protein sequence. In the independent testing dataset, ProteRNA has been able to deliver overall accuracy of 89.78%, MCC of 0.2628, F-score of 0.3075, and F_0.5_-score of 0.3546. It is anticipated that the prediction accuracy delivered by ProteRNA will continue to improve as the number of protein-RNA complexes deposited in the PDB continues to grow and the number of training samples that can be exploited continues to increase accordingly. Nevertheless, it is the computational biologists’ primary interest to develop more advanced prediction mechanisms. In this respect, we believe that, as the number of protein-RNA complexes deposited in the PDB increases, we can obtain more insights about the key physiochemical properties that play essential roles in protein-RNA interactions and then we will be able to develop more advanced prediction mechanisms accordingly. In addition, we will exploit the experiences learned in this study in order to design specific predictors for other families of proteins interacting with RNA. We believe that different families of proteins may have very different characteristics. Therefore, concerning a specific type of proteins, a specifically-designed predictor should be able to deliver superior performance in compared to a general-purpose predictor.

## Materials and methods

### Datasets

We used RB147 as the training dataset for predicting RNA-binding residues in a protein collected by Terribilini et al., containing 147 non-redundant protein chains with resolution better than 3.5 Å in the PDB solved by X-ray crystallography [31,40]. No two protein chains has a sequence identity greater than 30%. Based on the cut-off distance of 5 Å, a total of 32,324 amino acids are in RB147, which contains 6,157 RNA-binding residues and 26,167 non-binding residues. The list of PDB ids of the training dataset, RB147, is shown in Table 6(a).

**Table 6:** Datasets for ProteRNA

(a) Training dataset - RB147
1A34_A	1A9N_A	1APG_A	1ASY_A	1AV6_A	1B23_P	1B2M_A	1C0A_A
1DDL_A	1DFU_P	1DI2_A	1E8O_A	1EC6_A	1EIY_B	1F7U_A	1FEU_A
1FFY_A	1FJG_B	1FJG_C	1FJG_D	1FJG_E	1FJG_G	1FJG_I	1FJG_J
1FJG_K	1FJG_L	1FJG_M	1FJG_N	1FJG_P	1FJG_Q	1FJG_S	1FJG_T
1FJG_V	1G1X_A	1G1X_B	1G1X_C	1G2E_A	1GTF_Q	1H2C_A	1H3E_A
1H4S_A	1HQ1_A	1HRO_W	1I6U_A	1J1U_A	1J2B_A	1JBR_A	1JID_A
1K8W_A	1KNZ_A	1KQ2_A	1LAJ_A	1LNG_A	1M5O_C	1M8V_A	1M8X_A
1MZP_A	1N35_A	1N78_A	1NB7_A	1OOA_A	1PGL_2	1Q2S_A	1QF6_A
1QTQ_A	1R3E_A	1RMV_A	1RPU_A	1SDS_A	1SER_A	1SI3_A	1T0K_B
1TFW_A	1U0B_B	1UN6_B	1UVJ_A	1VFG_A	1VQO_1	1VQO_2	1VQO_3
1VQO_A	1VQO_B	1VQO_C	1VQO_D	1VQO_E	1VQO_G	1VQO_H	1VQO_I
1VQO_J	1VQO_K	1VQO_L	1VQO_M	1VQO_N	1VQO_P	1VQO_Q	1VQO_R
1VQO_S	1VQO_T	1VQO_U	1VQO_V	1VQO_W	1VQO_X	1VQO_Y	1VQO_Z
1W2B_5	1WNE_A	1WPU_A	1WSU_A	1WZ2_A	1Y69_8	1Y69_K	1Y69_U
1YVP_A	1YZ9_A	1ZH5_A	2A1R_A	2A8V_A	2ASB_A	2AVY_F	2AVY_U
2AW4_0	2AW4_1	2AW4_2	2AW4_3	2AW4_D	2AW4_E	2AW4_G	2AW4_H
2AW4_J	2AW4_L	2AW4_N	2AW4_P	2AW4_Q	2AW4_R	2AW4_S	2AW4_Y
2AW4_Z	2AZ0_A	2BGG_A	2BH2_A	2BTE_A	2BU1_A	2BX2_L	2CT8_A
2D3O_1	2D3O_S	2FMT_A					

(b) Independent Testing Dataset - RB33

1VS8_O	2D6F_D	2DB3_C	2DER_B	2DR2_A	2DU3_A	2F8S_A	2FK6_A
2G4B_A	2G8K_A	2GJE_A	2GJE_D	2GJW_C	2GTT_G	2GYA_3	2HVR_A
2HYI_D	2I82_C	2IPY_B	2IX1_A	2IY5_A	2J0Q_A	2JEA_A	2JEA_B
2NQP_B	2OZB_B	2PJP_A	2PY9_C	2Q66_A	2QAM_Z	2QBE_T	2QKK_F
2V47_C							

In order to evaluate prediction performance among different prediction models, we collected a new independent testing dataset by extracting all structures of Protein-RNA complexes from the PDB that were added after January 2006. Protein chains with a resolution better than 3.5 Å and sequence length of protein chain longer than 40 amino acids will be reserved. We then performed a redundancy reduction using BLASTclust [2] to ensure that none of the chains showed a sequence similarity of more than 30% within the dataset and also in the training dataset; therefore, 33 protein-RNA complexes were selected to create a dataset called RB33. The list of PDB ids in RB33 are shown in Table 6(b). Based on the cut-off distance of 5 Å, a total of 9,785 amino acids are in RB33, which contains 882 RNA-binding residues and 8,903 non-binding residues.

### Framework for prediction RNA-interacting residues

**Figure 3:**
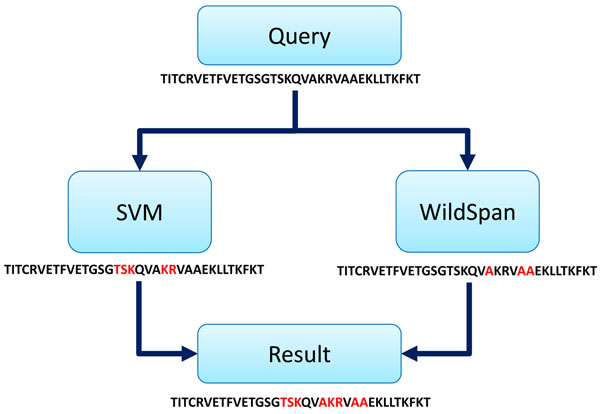
The overall framework of ProteRNA for predicting RNA-binding residues

**Figure 4:**
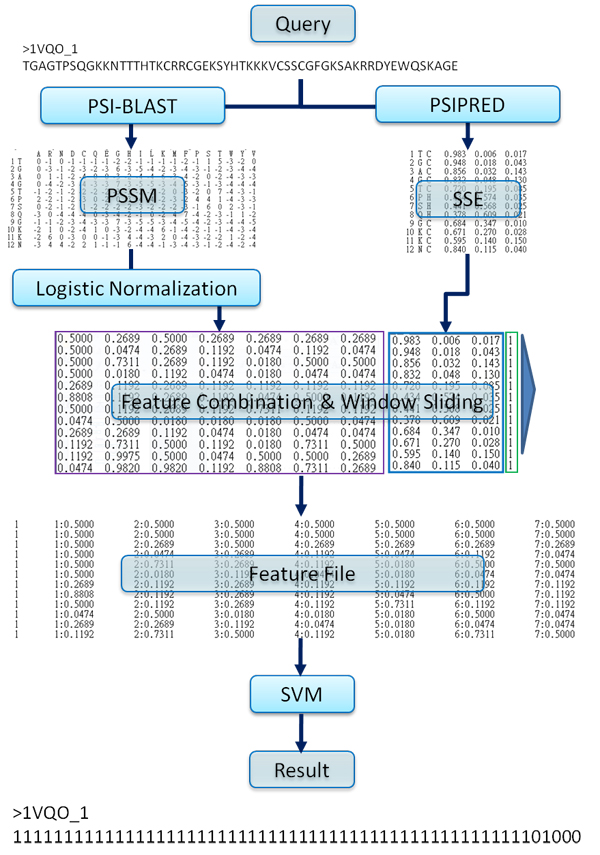
An outline of RNA-binding residue prediction by the SVM-based classifier.

Figure 3 presents the overall framework for predicting RNA-binding residues. In the overall framework, we combined SVM-based classifier and sequence conservation discovery by WildSpan to predict RNA-binding residues. For the SVM-based classifier (ProteRNA_SVM_), we have employed the LIBSVM package with the Gaussian kernel (software available at http://www.csie.ntu.edu.tw/~cjlin/libsvm). The model of the SVM has been generated based on the training data set derived by associating each residue in the training protein chains with the evolutionary profiles of the residue and its 22 neighboring residues (window size = 23) [43,44]. The evolutionary profile of a residue is in fact the vector corresponding to the residue in the position specific scoring matrix (PSSM) computed by the PSI-BLAST package [45] with three iterations (blastpgp -j 3) against with NCBI non-redundant reference sequence database (ftp://ftp.ncbi.nih.gov/blast/db/). The normalization function for PSSM features is defined as follow:

where *x* is the entry value in 20xN matrix of PSSM (N is sequence length of a protein). With the consideration of structure information, we also used secondary structure information predicted by PSIPRED; the predicted secondary structure information consists of three probability values that represent helix, sheet and coil respectively (*e.g.* (H, E, C) = (0.75, 0.25, 0.25)). In addition, each residue was labelled based on whether it is involved in binding with the RNA or not. Therefore, for each residue in a protein sequence, we construct a 23 * 24 = 552 dimensional feature factor (window size = 23, feature size = 24); the 24 dimensions include 20 features from PSSM, 3 features from PSIPRED and a boundary flag. As shown in Figure 4, the detail data flow and feature vector preparation for SVM-based classifier is addressed. The best parameters selected for predicting RNA-binding residues is decided by 5-fold cross-validation.

In the part of WildSpan (ProteRNA_WildSpan_), for protein-based mining suggested by the authors, at most 150 unique homologous proteins with sequence identity ranged from 30% to 90% are required by searching against Swiss-Prot sequence database with PSI-BLAST (blastpgp –j 6). Then we applied default parameter to obtain patterns by WildSpan. WildSpan can’t generate any pattern if there are not enough homologous proteins selected from Swiss-Prot protein sequence database or too similar homologous proteins.

### Significance and performance evaluation

The predictions made for the testing instances are compared with the defined class labels (binding or non-binding) to evaluate the predictor. The accuracy is defined as

where TP is the number of true positives (binding residues with positive predictions); TN is the number of true negatives (non-binding residues with negative predictions); FP is the number of false positives (non-binding residues but predicted as binding residues) and FN is the number of false negatives (binding residues but predicted as non-binding residues). Matthew's correlation coefficient (MCC) is defined as follows:

MCC is used to measure prediction performance with the consideration of both under- and over-predictions, where MCC = 1 denotes a perfect prediction, MCC = 0 indicates a completely random assignment, and MCC = -1 means a perfectly reverse correlation.

Since the data for RNA-binding residue prediction is skewed, so-called class imbalanced data, the accuracy alone may be misleading. The predictor can achieve 85% accuracy by simply predicting all residues as negative for datasets where the positive to negative sample ratio is 1:10. Therefore, prediction performance on positive class and negative class should be assessed individually. Metrics of the specificity and sensitivity can help predictors to know their prediction performance on positive and negative samples respectively. The sensitivity is used to measure the prediction capability of positive samples; the specificity is used to measure the prediction capability of negative samples. Specificity and sensitivity are defined as follows:

In addition, precision and F_β_-score are also defined as follows:

Precision is used to assess prediction power on positive class. F-score (F_1_-score) is the harmonic mean of precision and Sensitivity if β = 1. F_0.5_-score weights precision twice as much as sensitivity if β = 0.5.

## List of abbreviations

RBP: RNA-binding protein, RBD: RNA-binding domain; RNP: Ribonucleoprotein particle; PSSM: Position-specific scoring matrix; NDB: Nucleic Acid Database; PDB: Protein Data Bank; SVM: Support vector machine.

## Competing interests

The authors declare that they have no competing interests.

## Authors' contributions

YFH and LYC developed and implemented the overall framework and drafted the manuscript; CCH provided valuable suggestion on experiments based on his previous works on predicting DNA-binding residues. CKH conceived of the study and participated in its design and coordination and helped to draft this manuscript. All authors have read and approved the final manuscript.
